# A novel approach for safe and automated implementation of far ultraviolet-C light decontamination in clinical areas

**DOI:** 10.1017/ash.2024.388

**Published:** 2024-09-09

**Authors:** Samir Memic, Jennifer L. Cadnum, Andrew Osborne, William A. Rutala, Curtis J. Donskey

**Affiliations:** 1 Department of Systems Biology, Case Western Reserve University School of Medicine, Cleveland, OH, USA; 2 Research Service, Louis Stokes Cleveland VA Medical Center, Cleveland, OH, USA; 3 Department of Medicine, Case Western Reserve University School of Medicine, Cleveland, OH, USA; 4 Statewide Program for Infection Control and Epidemiology, University of North Carolina School (UNC) of Medicine, Chapel Hill, NC, USA; 5 Division of Infectious Diseases, UNC School of Medicine, Chapel Hill, NC, USA; 6 Geriatric Research, Education, and Clinical Center, Louis Stokes Cleveland VA Medical Center, Cleveland, OH, USA

## Abstract

A novel wall-mounted far ultraviolet-C (UV-C) light technology providing automated delivery of far UV-C only when people are not present reduced methicillin-resistant *Staphylococcus aureus* in a patient room and equipment room. The safety feature that discontinues far UV-C output when people are detected was effective in preventing far UV-C exposure.

## Introduction

Far ultraviolet-C (UV-C) light (200–230 nm) has been proposed as a technology that could provide continuous, safe air and surface decontamination in occupied spaces.^1–3^ There is evidence that far UV-C doses within threshold limit values proposed by the American Conference of Governmental Industrial Hygienistsand the International Commission on Non-Ionizing Radiation Protection may be safe.^1,4^ However, safety evaluations have involved animal or *in vitro* skin models with only preliminary reports in exposed humans.^1,4–6^

One innovative approach that might allow safer use of UV-C technologies in clinical areas could be addition of motion detectors with discontinuation of UV-C delivery when motion is detected.^7,8^ Here, we evaluated the efficacy and safety of a novel wall-mounted, automated far UV-C light technology that detects the presence of people and only delivers far UV when people are not present.^3^

## Methods

### Description of the far UV-C light technology

The wall-mounted far UV-C technology (Mynatek, Inc., Oakland, CA) uses 3 krypton-chloride excimer lamps emitting a primary wavelength of 222 nm with a field of illumination of ∼60° per lamp.^3^ An adjustable arm allows adjustment of lamp position and orientation. For this study, the devices were mounted on posts adjacent to the wall. Two devices are recommended for a typical patient room. The device includes proprietary sensors that detect people within the field of illumination, including individuals remaining motionless. The device was programmed to automatically discontinue far UV-C light delivery whenever people were detected and to automatically resume delivery when they moved outside the area of exposure.

### Evaluation of the far UV technology in a patient room and equipment room

Testing was conducted in an unoccupied 38.0 m^3^ patient room. Initial testing demonstrated that positioning of the devices on opposite sides of the bed versus on the same side resulted in improved far UV-C delivery with no unexposed shaded areas based on colorimetric indicator results. The supplemental material shows a picture of the device, dimensions of the patient room, far UV-C doses and MRSA log_10_ reductions at the test sites, pictures of the test rooms, and MRSA reductions in the patient bathroom. Based on the initial results, testing was conducted with 2 devices positioned at a height of 2 m at opposite sides of the room with the bed between the devices. A 45-minute continuous exposure was chosen based on previous evidence that vegetative organisms on steel disk carriers were reduced by >3 log_10_ after 45 minutes.^3^ For 5 sites located 1.5–2 m from the nearest device, efficacy against methicillin-resistant *Staphylococcus aureus* (MRSA) was tested using a modification of the American Society for Testing and Materials (ASTM) standard quantitative disk carrier test method (ASTM E 2197-02) with 5% fetal calf serum as soil load.^9^ MRSA was chosen for testing because it is a common healthcare-associated pathogen. Experiments were performed in triplicate. A reduction of ≥3 log_10_ in comparison to untreated controls was considered effective.^10^

Additional testing was conducted in 35.2 m^3^ equipment storage room with 2 devices placed on opposite sides of the room. A workstation-on-wheels, portable vital signs unit, and wheelchair were inoculated on 2.5 cm diameter circular areas with 10 µL containing 10^6^ colony-forming units (CFU) of MRSA in 5% fetal calf serum; this method was used to assess reductions on real-world devices. The test sites ranged from 1.5 to 2.2 m from the nearest device and included sites directly facing the far UV-C lamps and at the side of the devices. After 45 minutes and 4 hours of exposure, inoculated sites were sampled with premoistened cotton-tipped swabs. The swabs were processed to quantify MRSA.^3^ Testing was completed in triplicate. Log_10_ reductions were calculated in comparison to untreated control surfaces.

### Evaluation of the safety feature that discontinues far UV-C output when people are present

To assess the feature that discontinues far UV delivery when people are present, we conducted 20 structured assessments with 1 device in an open area. For these assessments, the individual walked toward the device from 20 different angles ranging from in front of the device to 180-degree angles from the side until the device turned off based on visual assessment (i.e., the lamps emit visible light when on); the individual stood motionless for 5 minutes prior to moving out of range of the detection system. The purpose of the structured assessments was to determine if the devices consistently detect people in locations where far UV-C is delivered, remain off if a person remains motionless, and turn back on after the person exits the zone of delivery. A radiometer (UIT2400 Handheld Light Meter for 222 nm, Ushio America, Cypress, CA) was used to measure irradiance at different locations relative to the device with the device on to determine if people would be exposed to far UV-C with the lamps operating. To further assess far UV-C exposure, research personnel conducting the experiments wore 222UVC Dots colorimetric indicators (Intellego Technologies, AB Gothenburg, Sweden) that detect doses ranging from 5 to 150 mJ/cm^2^.

## Results

### Evaluation of the far UV-C technology in a patient room and equipment room

**Figure 1.**
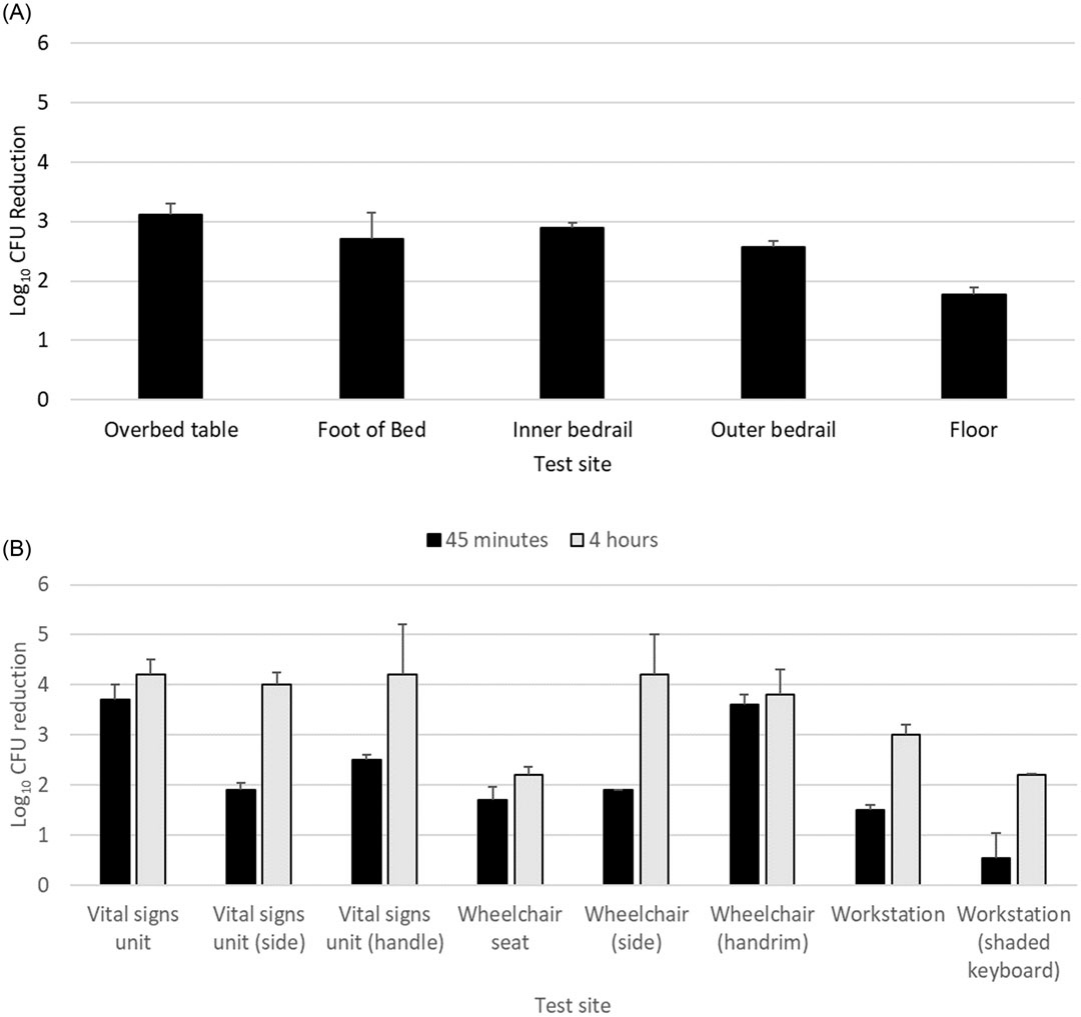
Reductions in methicillin-resistant *Staphylococcus aureus* (MRSA) in an unoccupied patient room with a 45-minute far ultraviolet-C exposure time (A) and in a portable equipment room with 45-minute and 4-hour exposure times (B). The workstation was a portable workstation-on-wheels. Error bars show standard error.

After 45 minutes of exposure, MRSA was reduced by ≥1.7 log_10_ at all sites in the patient room (Figure 1). In the equipment room, 45 minutes of exposure reduced MRSA by ≥1.6 log_10_ CFU at each site except a partially shaded keyboard on the workstation-on-wheels (.5 log_10_ reduction); after 4 hours exposure, MRSA was reduced by ≥3 log_10_ at 6 of 8 test sites.

### Evaluation of the safety feature that discontinues far UV-C output when people are present

**Figure 2.**
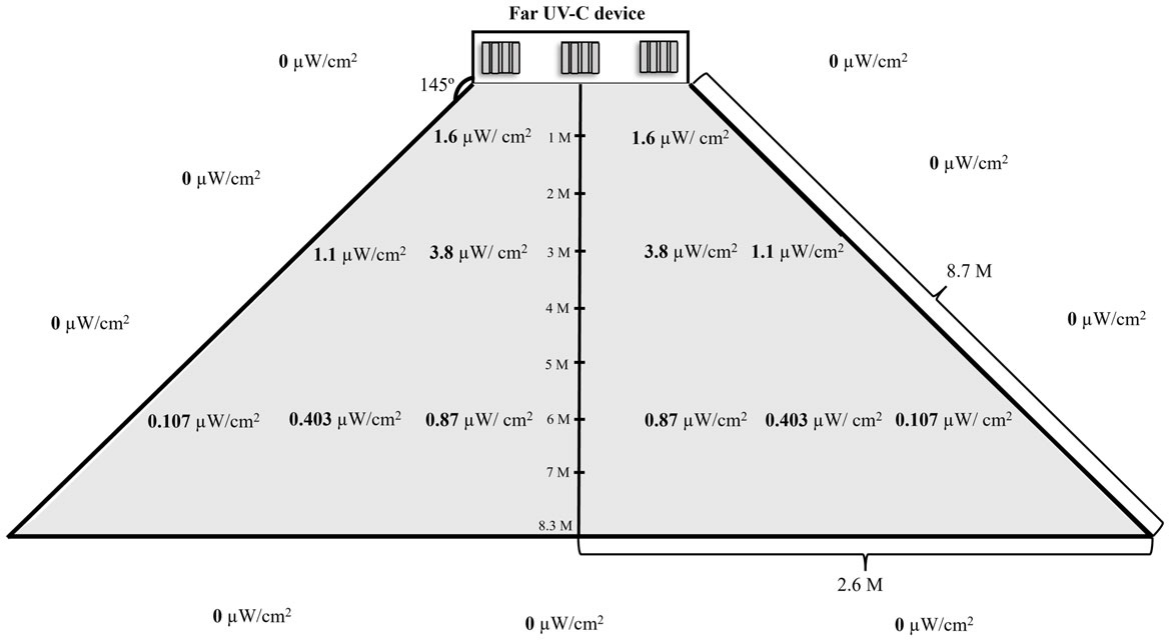
Illustration of a far ultraviolet-C (UV-C) device showing irradiance readings and the area where the device turned off and stayed off upon entry of a person into zone of detection which is the gray shaded area. The irradiance readings with the device on with no one in the zone of detection are shown; far UV-C light was not detected outside the shaded area while the device was on. Readings of 0 indicate no detection of far UV-C above baseline negligible levels measured with the device off. The device resumed far UV-C delivery 30 seconds after a person exited the zone of detection.

Figure 2 provides an illustration of the area where a far UV-C device turned off upon entry of a person into the vicinity of the device (shaded in gray). Once the device turned off, it remained off while people stood motionless in the shaded areas and turned back on 30 seconds after they exited. Based on irradiance readings, far UV-C light was not detected outside the shaded area while the device was on. During the experiments, colorimetric indicators worn by research personnel indicated no detectable exposure to far UV-C (limit of detection, 5 mJ/cm^2^).

## Discussion

We demonstrated that a wall-mounted technology modified to provide automated delivery of far UV-C only when people are not present was effective in reducing MRSA in a patient room and equipment room. The log_10_ reductions achieved at the test sites varied likely due to factors such as variations in the far UV-C dose delivered, orientation of the test organisms versus the light source, and partial shading. However, >3 log_10_ reductions were achieved on 6 of the 8 inoculated device sites after 4 hours of exposure. The safety feature that discontinues far UV-C output when people are present was effective in preventing exposure to far UV-C light. Our findings suggest that the far UV-C technology could be used to provide automated, intermittent surface decontamination in clinical areas during periods when people are not present.

This study has some limitations. The study was conducted in unoccupied areas and only 1 pathogen was studied. Only 45-minute and 4-hour exposures were tested. Longer exposure times would be anticipated in areas such as equipment rooms that are occupied infrequently. Future studies are needed to determine if use of the technology will reduce healthcare-associated infections.

## Supporting information

Memic et al. supplementary material Memic et al. supplementary material

## Supplementary material

For supplementary material accompanying this paper visit http://doi.org/10.1017/ash.2024.388. click here to view supplementary material
