# Supplementary material for: A novel approach for safe and automated implementation of far ultraviolet-C light decontamination in clinical areas
Source: Antimicrob Steward Healthc Epidemiol. 2024 Sep 9;4(1):e123. doi: 10.1017/ash.2024.388 (PMC11384163; doi:10.1017/ash.2024.388)

**Supplementary material**

**Title: A novel approach for safe and automated implementation of far ultraviolet-C light decontamination in clinical areas**

**Figure 1.** Picture of the wall-mounted device showing the 3 krypton-chloride excimer lamps and adjustable arm that can be used to adjust the position of the lamps.

**
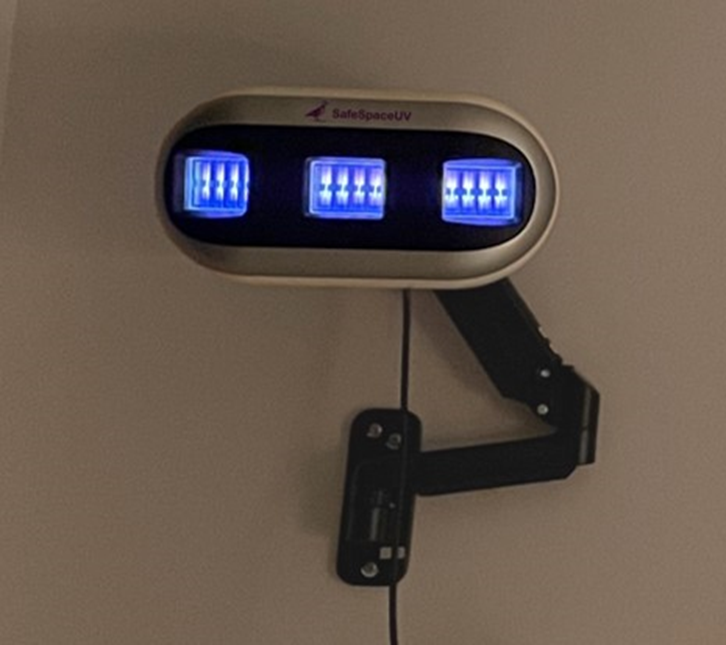
**

**Figure 2.** Patient room dimensions without the far UV-C devices


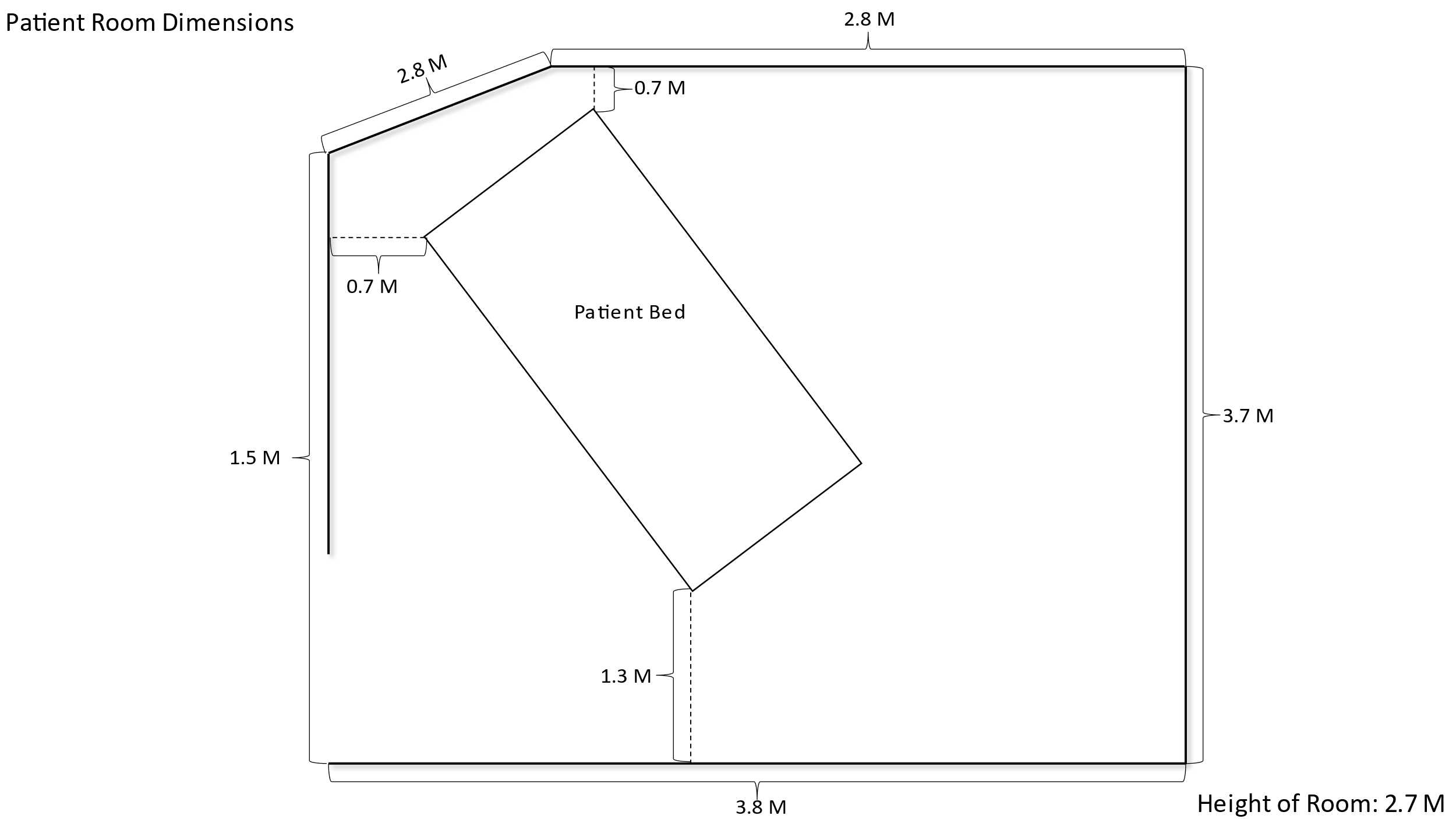


**Figure 3**. Far ultraviolet-C (UV-C) doses based on colorimetric indicator readings in an unoccupied patient room with 2 far UV-C devices positioned either along 1 wall (A) or at opposite sides of the room with the bed midway between the lamps (B) and reductions in methicillin-resistant *Staphylococcus aureus* (MRSA) after 45 minutes of exposure (C). Doses of far UV-C measured using colorimetric indicators are shown in parentheses. Error bars show standard error.

**
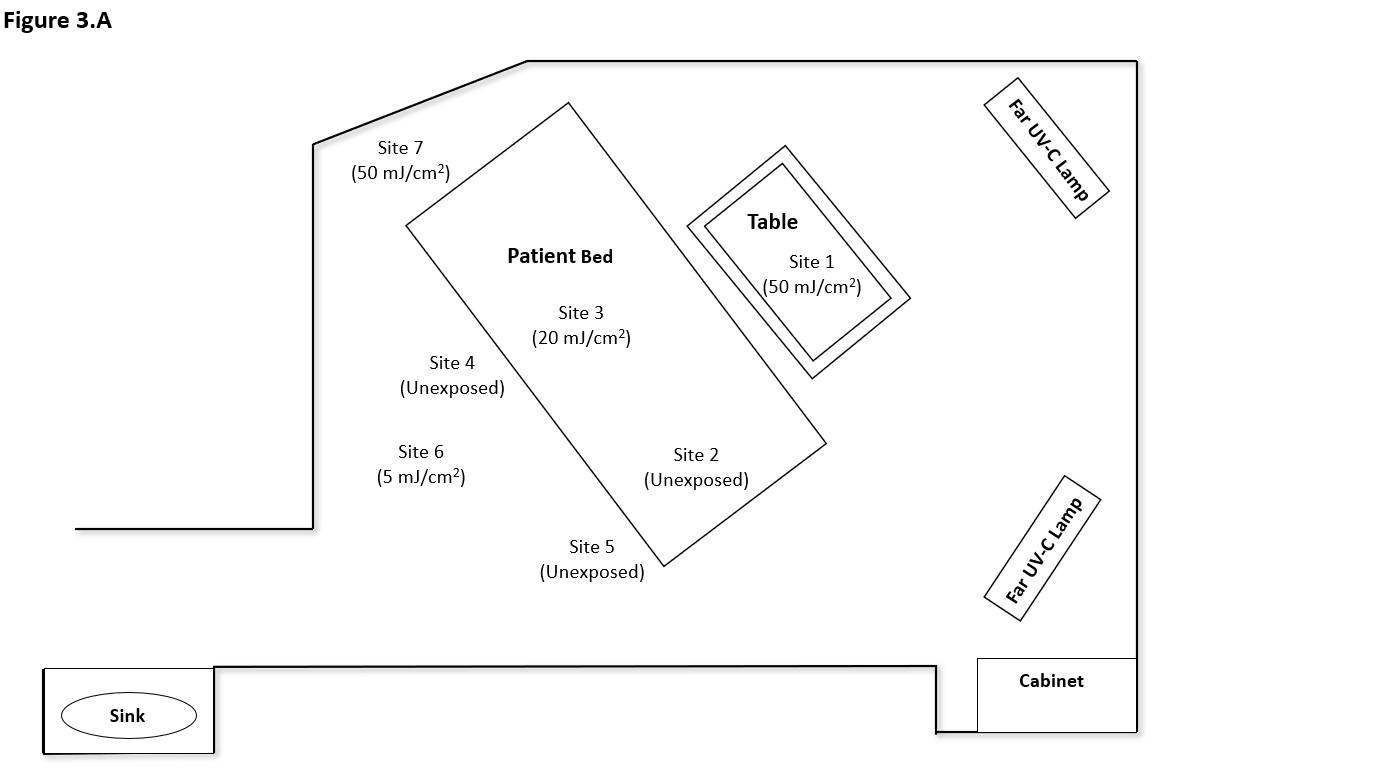
**

**
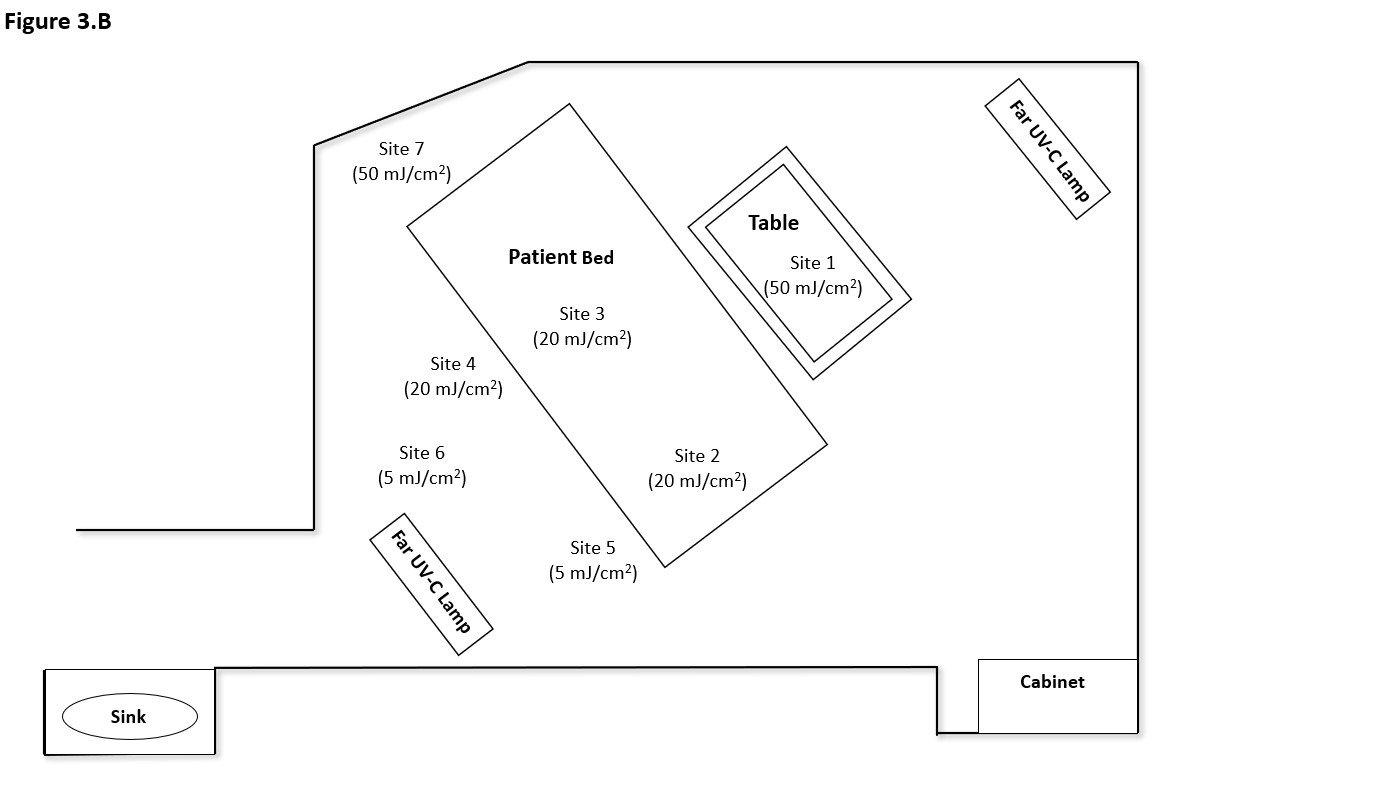
**

**Figure 3.C**

**
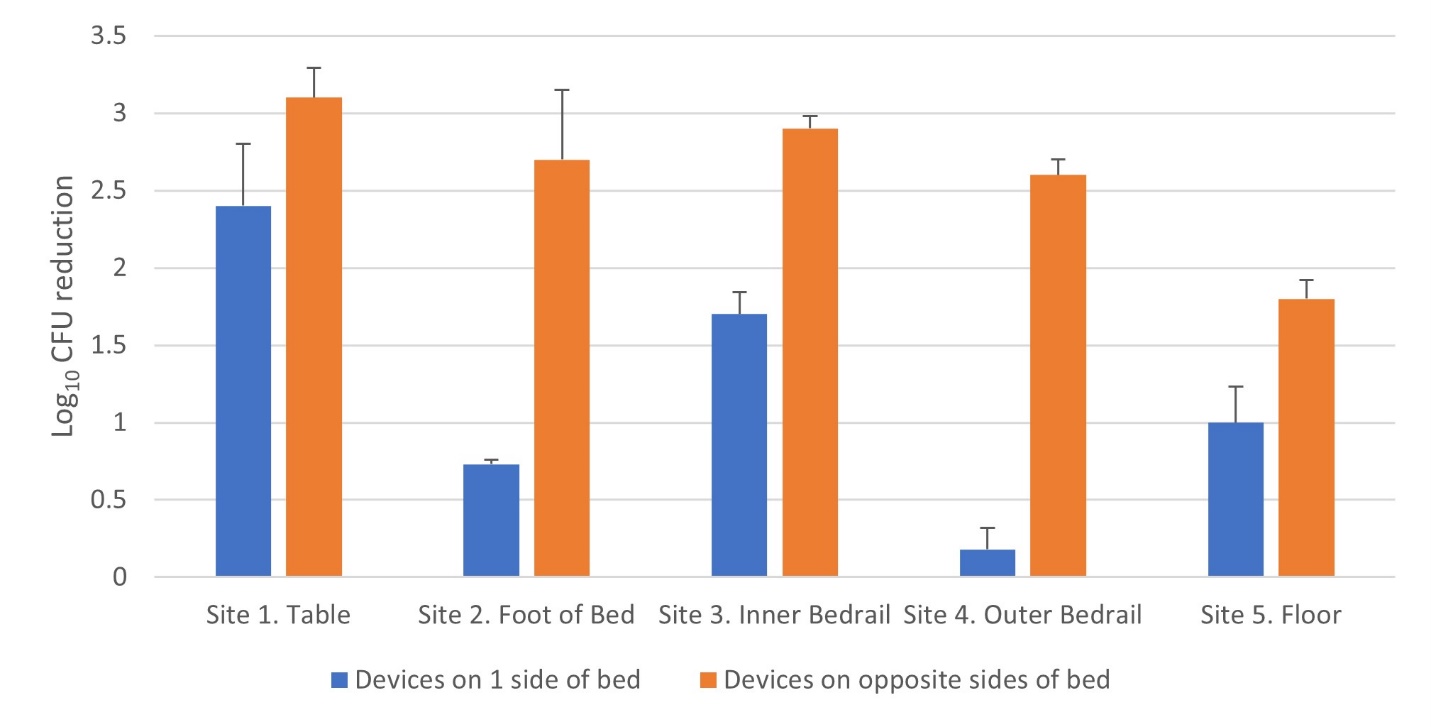
**

**Figure 4.** Reductions in methicillin-resistant *Staphylococcus aureus* (MRSA) after 45 minutes of exposure in a patient bathroom. Doses of far UV-C measured using colorimetric indicators are shown in parentheses. Error bars show standard error.

**Figure 5.** Pictures of the patient room and equipment room with the devices in place

1. Patient room with 2 far UV-C devices positioned in parallel along 1 wall


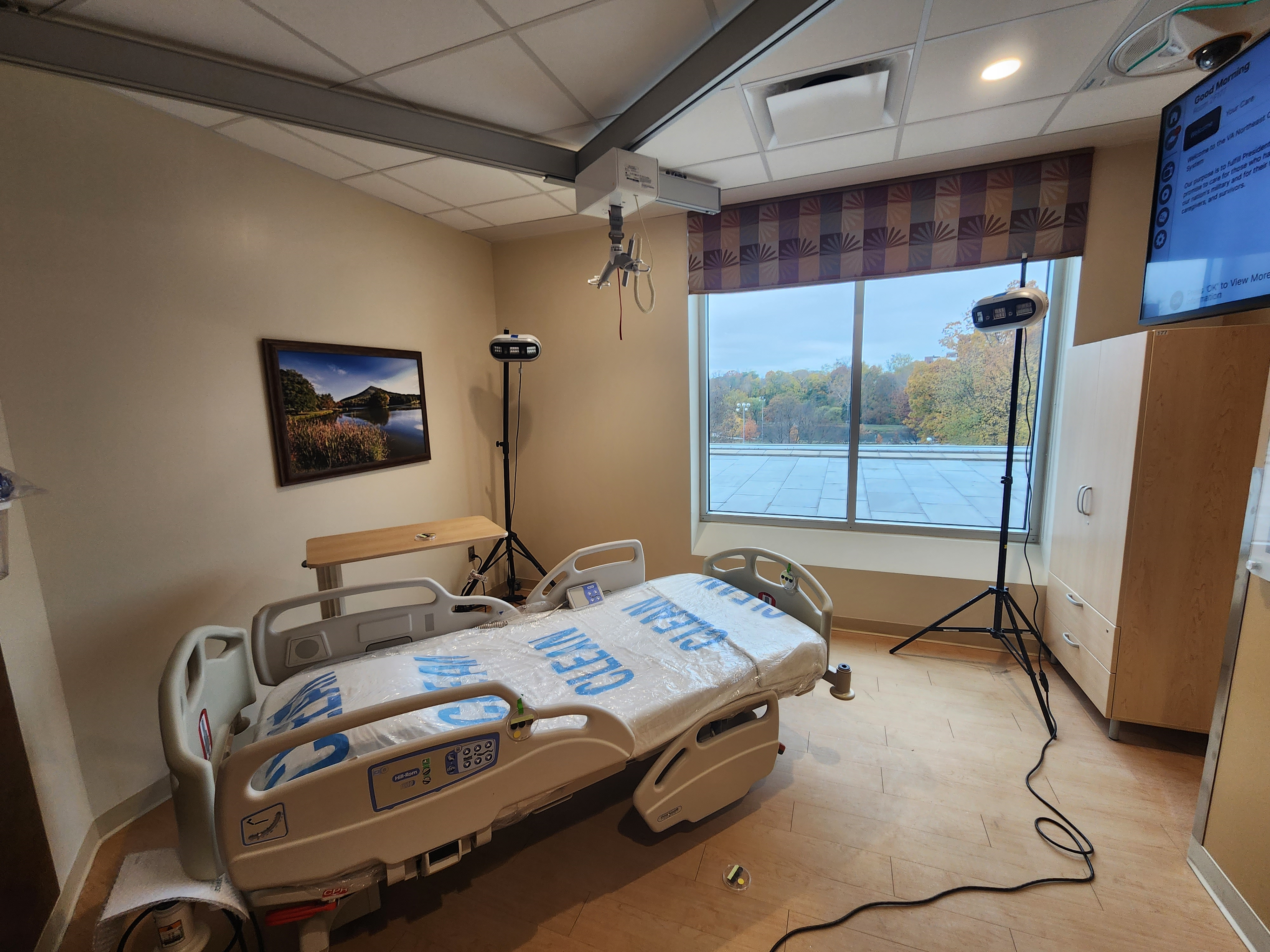


1. Patient room with 2 devices positioned at opposite sides of the room on each side of the bed


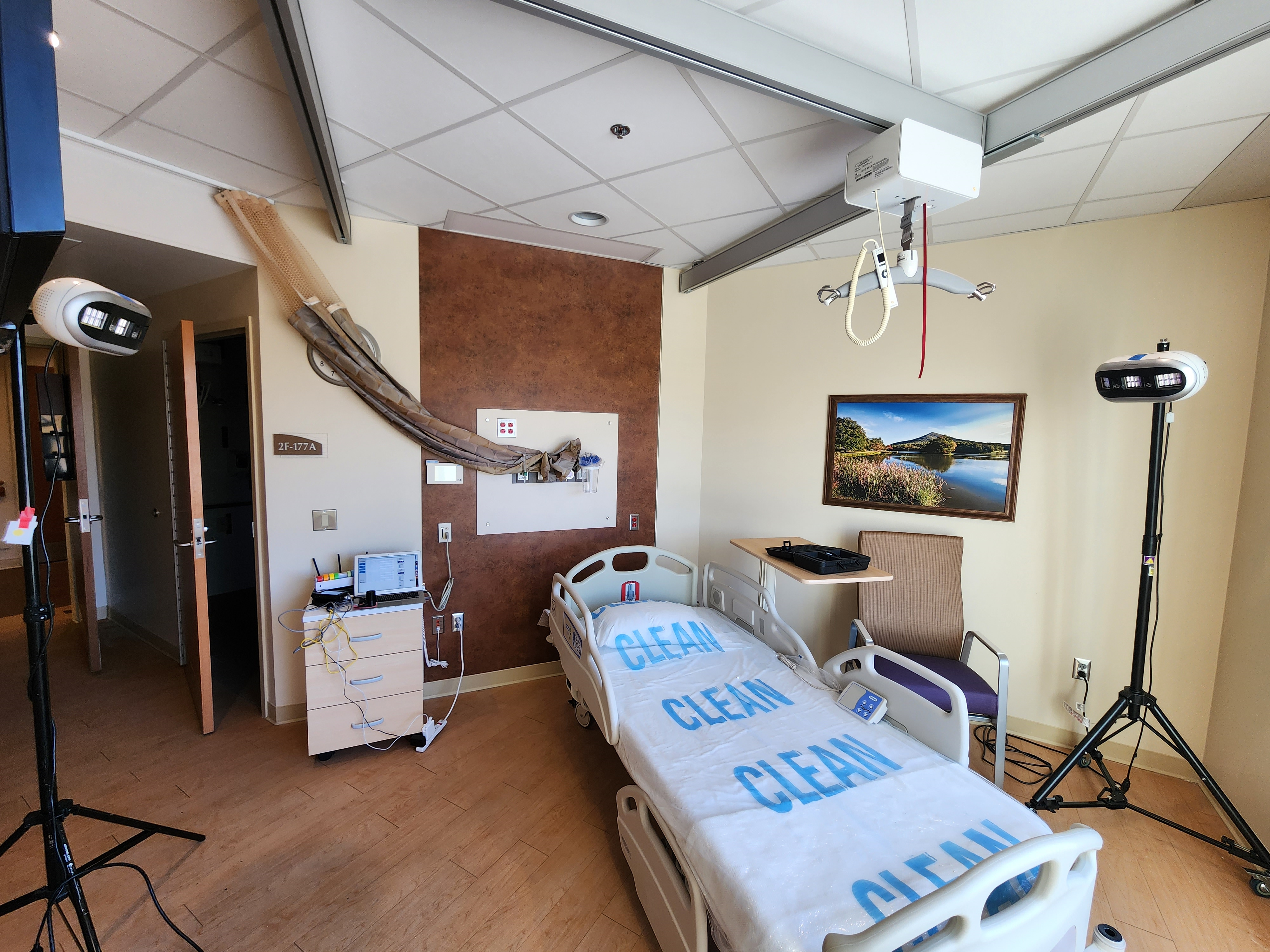


1. Equipment room
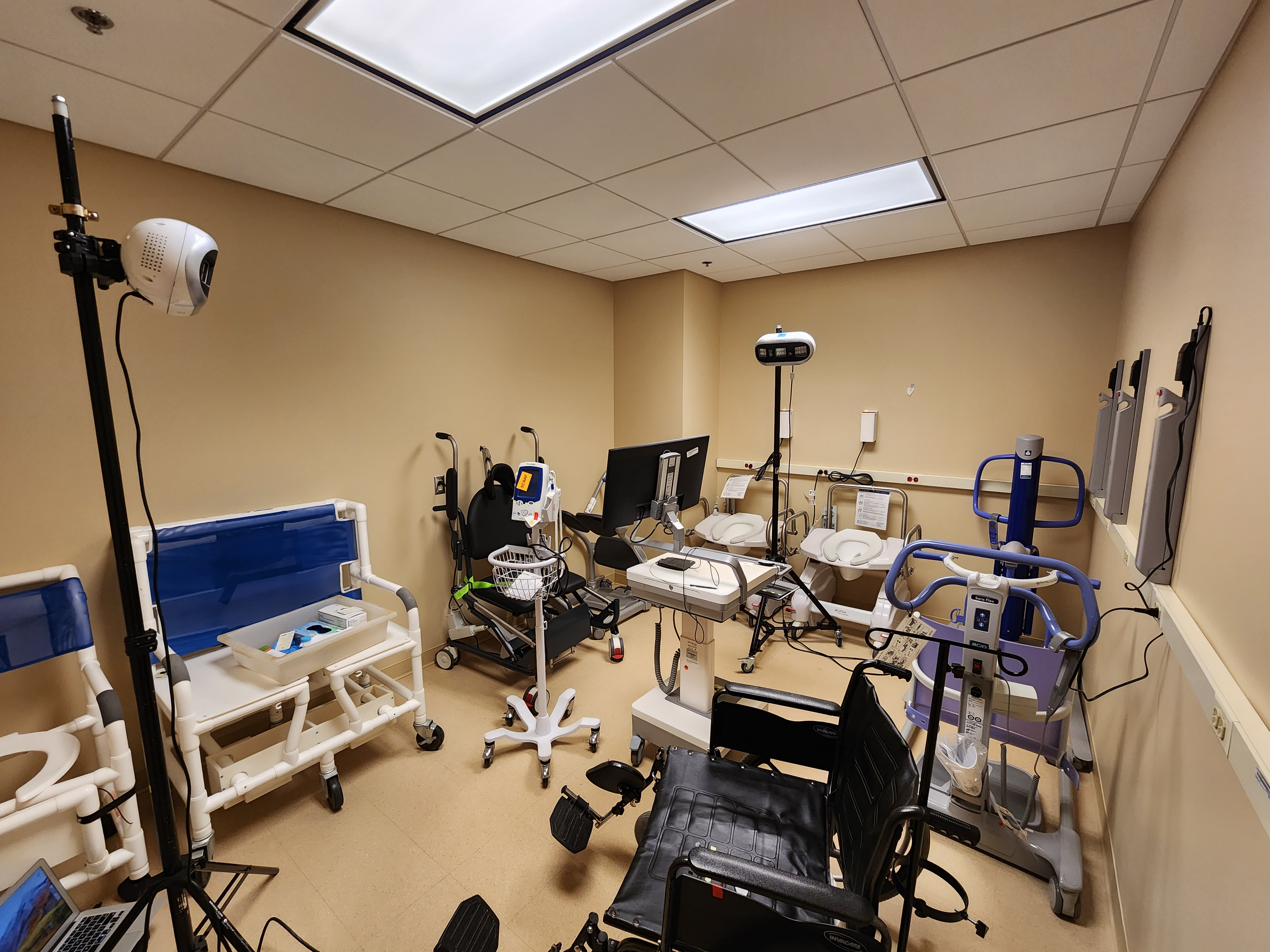

Supplement: Memic et al. supplementary material [file S2732494X24003887sup001.docx]
